# Comparative Transcriptome Analysis of Two Root-Feeding Grape Phylloxera (*D. vitifoliae*) Lineages Feeding on a Rootstock and *V. vinifera*

**DOI:** 10.3390/insects11100691

**Published:** 2020-10-12

**Authors:** Stefania Savoi, Markus W. Eitle, Harald Berger, Manuel Curto, Harald Meimberg, Michaela Griesser, Astrid Forneck

**Affiliations:** 1 Department of Crop Sciences, Institute of Viticulture and Pomology, University of Natural Resources and Life Sciences Vienna, Konrad Lorenz Straße 24, 3430 Tulln, Austria; savoi.stefania@gmail.com (S.S.); markus.eitle@boku.ac.at (M.W.E.); harald.berger@boku.ac.at (H.B.); michaela.griesser@boku.ac.at (M.G.); 2 Institute Agro, UMR AGAP, Montpellier University, CIRAD, INRAe, Via P. Viala, 34060 Montpellier, France; 3 Department of Integrative Biology and Biodiversity Research, Institute for Integrative Nature Conservation Research, University of Natural Resources and Life Sciences Vienna, Gregor-Mendel-Str. 33, 1180 Vienna, Austria; manuel.curto@boku.ac.at (M.C.); meimberg@boku.ac.at (H.M.)

**Keywords:** RNA-sequencing, grape phylloxera, insect biotypes, effectors, host–parasite interaction, insect chemoreception

## Abstract

**Simple Summary:**

Grape phylloxera is an American native insect pest that caused heavy damages to the vineyards worldwide since its spreading to wine regions since the 1850s. This insect, able to feed on leaves and roots, induces plant galls and manipulates the grapevine physiology leading to plant damage and may cause plant death. The most successful treatment was the use of mostly partially resistant rootstocks. The degree of resistance is affected by environment, grapevine management and the insect biotype. In this study, we analyse the interaction of insect biotypes feeding on particular host plants. Therefore we evaluated the gene expression of Phylloxera feeding on a susceptible host versus feeding on a rootstock in two different developmental stages. We discovered (mainly in advanced insect developmental stages) genes expressed in higher proportion in one insect compared to the other. These genes related to chemosensory; in plant physiology manipulation and root deformation and insect digestive traits may play a role in the plant-insect interaction determining plant resistance in response to the pest attack.

**Abstract:**

Grape phylloxera is one of the most dangerous insect pests for worldwide viticulture. The leaf- and root-galling phylloxerid has been managed by grafting European grapevines onto American rootstock hybrids. Recent reports pinpoint the appearance of host-adapted biotypes, but information about the biomolecular characteristics underlying grape phylloxera biotypisation and its role in host performance is scarce. Using RNA-sequencing, we sequenced the transcriptome of two larval stages: L1 (probing) and L2-3 (feeding) larvae of two root-feeding grape phylloxera lineages feeding on the rootstock Teleki 5C (biotype C) and *V. vinifera* Riesling (biotype A). In total, 7501 differentially expressed genes (DEGs) were commonly modulated by the two biotypes. For the probing larvae, we found an increased number of DEGs functionally associated with insect chemoreception traits, such as odorant-binding proteins, chemosensory proteins, ionotropic, odorant, and gustatory receptors. The transcriptomic profile of feeding larvae was enriched with DEGs associated with the primary metabolism. Larvae feeding on the tolerant rootstock Teleki 5C exhibited higher numbers of plant defense suppression-associated DEGs than larvae feeding on the susceptible host. Based on the identified DEGs, we discuss their potential role for the compatible grape phylloxera–*Vitis* interaction belowground. This study was the first to compare the transcriptomes of two grape phylloxera lineages feeding on a tolerant and susceptible host, respectively, and to identify DEGs involved in the molecular interaction with these hosts. Our data provide a source for future studies on host adaptation mechanisms of grape phylloxera and help to elucidate grape phylloxera resistance further.

## 1. Introduction

Agricultural food production has continuously been challenged by the advent of newly host-adapted and more aggressive insect biotypes infesting crop species or specific plant genotypes, frequently promoted by predominant agricultural monocultivation, excessive pesticide use, and ongoing climate change, which favored rapid insect development and its reproduction rates [1,2,3].

Grape phylloxera (*Daktulosphaira vitifoliae* fitch) is an aphid-like insect belonging to the Phylloxeridae family within the Hemiptera order [4,5,6]. Since its introduction from North America into Europe in the late 19th century, grape phylloxera has become one of the most dangerous pest species for worldwide viticulture [7]. The insect feeds monophagously on *Vitis* spp. host plants by imbibing the cellular content retrieved from the nutritive organoid root or histoid leaf galls [8,9,10]. Grape phylloxera root infestation manipulates the host vine physiology by modulating the water, mineral, and assimilate transport pathways [11,12,13], interfering with host plant defense mechanisms [14,15,16,17,18] and facilitating secondary root infections by phytopathogenic soil-borne microorganisms [19], altogether leading to host plant damage or even vine death depending on concomitant biotic and abiotic environmental factors [20,21]. Motile grape phylloxera L1 larvae move and probe *Vitis* spp. root tips in order to select suitable host plants and feeding sites for the root gall establishment [22]. Although this interaction is well studied, many aspects of the molecular background of grape phylloxera–root interaction remain unknown. The larval ability to overcome primary and secondary host defense barriers plays a critical role early in the compatible host–parasite interaction [17,23,24].

The most successful treatment against grape phylloxera is the grafting of European grapevine scion cultivars (*V*. *vinifera*) onto American rootstock hybrids derived from accessions of, e.g., *V. berlandieri, V. riparia*, and *V. rupestris* [25]. Other treatments (e.g., pesticides, quarantine) do not provide sustainable long-term solutions without economic and ecological costs. The majority of rootstocks used in modern viticulture are ranked tolerant against grape phylloxera, thus hosting grape phylloxera populations without host damage [20]. Reports of grape phylloxera root infestations leading to crop loss and significant vine damage are increasing worldwide [26,27,28], partially explained by the evolution and spread of host-adapted grape phylloxera biotypes [29,30]. A classification system for grape phylloxera defines seven biotypes (A-G), based on insect performance (e.g., life table parameters), host plant responses (e.g., gall numbers), and the feeding tissue (leaf versus root) [31]. Biotype A shows a superior insect performance and root-galling ability on own-rooted *V. vinifera* plants [30,32], while biotype C performs best on roots of rootstock hybrids [33,34].

Although the morphological modifications during grape phylloxera root gall formation are comparably well studied [8,9,13,16,17,24,35,36,37], the underlying biomolecular basis of root-feeding grape phylloxera biotypes is far from clear. Work employing molecular markers failed to identify biomarkers linked to host preference, e.g., [25,28,38,39] other than leaf feeding in native habitats.

The Grape Phylloxera Genome Sequencing Initiative [5] induced a series of transcriptomic and effector studies to elucidate the complex interactome of this pest. The first comparative transcriptome analysis published showed that root- and leaf-feeding grape phylloxera stages exhibit major intraspecific changes in transcriptomic profiles related to different lifestyles [40]. Studies analyzing genes underlying effector proteins [41,42,43,44] have been very useful in understanding the complexity of the grape phylloxera–*Vitis* interaction. As a cyclical parthenogenetic monophagous and gall-feeding insect that cohabitates with technologically altered host plants (grafts) in extremely diverse habitats (introduced and native), changes in performance and genetic structures among phylloxera populations are likely [26,45,46]. Previously, transcriptomic and effector studies focused on feeding and developmental life stages [40], geographical or phylogenetic distribution [43,44,47], or genomic functional observations [41,42]. Newer studies on grape phylloxera feeding sites (leaf and root galls) show the massive impact on the metabolism of the host plants [10,13,48]. Although host-plant effects seem evident by host performance studies (reviewed in [20,26]), data on molecular mechanisms targeting the effector profiles comparing host-adapted phylloxera populations are missing.

Here, we present the first transcriptomic comparison between two root-feeding grape phylloxera lineages feeding on different host plants ranked as tolerant (Teleki 5C) and susceptible (*V. vinifera* cv. Riesling). Transcriptomic profiles of L1 versus L2-3 larval stages were analyzed to compare probing versus feeding and developing processes on the tolerant and susceptible host. We report the identification of DEGs putatively (1) involved in chemoreception and host infestation processes of L1 larvae and (2) DEGs coding for putative effector candidates secreted by L2-3 larvae, some of which are discussed to play a role for the grape phylloxera–*Vitis* interaction belowground.

## 2. Materials and Methods

### 2.1. Insect and Plant Material

**Figure 1:**
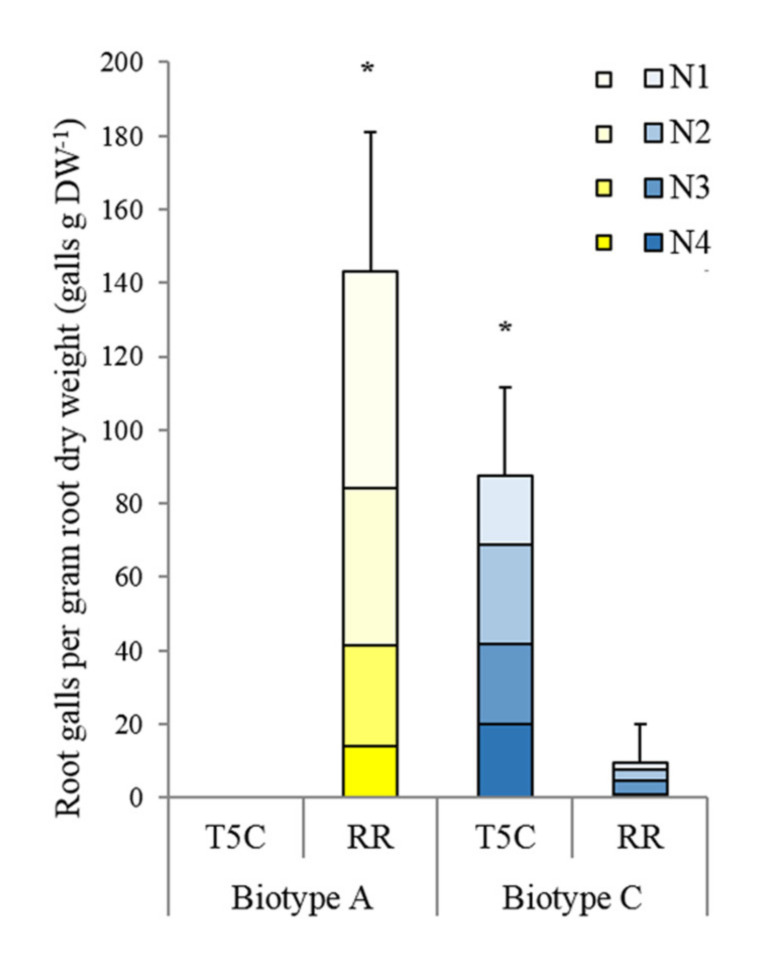
Average number of root galls (galls gDW^−1^) formed by grape phylloxera biotype A and C on roots of potted *V. vinifera* L. Riesling (RR) and the rootstock Teleki 5C (*V. berlandieri* × *V. riparia*) (T5C). Galls were categorized by size: N1 < 0.3 cm, N2 0.3 cm–0.6 cm, N3 > 0.6 cm, and N4 = inseparable galls. Error bars represented standard deviations of the sum of galls (N1–N4). Asterisks illustrated significant differences obtained by Mann–Whitney U testing with *p* < 0.05 comparing the sum of galls (N1–N4) produced by each biotype on the host versus the non-host.

Grape phylloxera eggs were taken from two grape phylloxera single founder lineages propagated in vitro on excised *Vitis* roots of either *V. vinifera* Riesling or Teleki 5C (*V. berlandieri* x *V. riparia*) [49] and determined to be biotypes A and C, respectively (Figure 1). Grapevine single bud cuttings were derived from dormant one-year-old canes, taken from *V. vinifera* Riesling (Gm 239) and Teleki 5C (*V. berlandieri* x *V. riparia*, Gm 6-52). These plants were grown at the research vineyards of the Institute of Viticulture and Pomology of BOKU University in Tulln Austria. Single eye cuttings were dipped in a rooting solution (0.1% IBA and 0.07% NAA), potted in 6 × 6 cm Jiffy pots, and cultivated under greenhouse conditions to promote root and shoot development for 1.5 months.

### 2.2. Insect Samples for RNA Extraction

For RNA-sequencing, 45 pre-rooted vines of Riesling and Teleki 5C were planted in six growth containers, each one containing 15 vines, filled with a clay:perlite substrate (1:1). Each container was inoculated with 200 eggs of two single founder lineage cultivated on either *V. vinifera* Riesling (named biotype A) and cultivated on the rootstock Teleki 5C (named biotype C). Insects hatched and developed on the host roots in a climate chamber set to 25 ± 3 °C, 45 ± 5% rH, 16-h photoperiod. Previous grape phylloxera bioassay studies showed that both changes of the gall physiology and the insect life stage are tightly correlated with feeding time: larval stages L2, L3, and L4 correspond to 2–7, 8–13, and >14 days after infestation (dai), while the adult stage (A) is characterized by oviposition [4]. After 55 days (2nd insect generation), 80 L2-3 larvae per sample (N = 3) were detached from the root galls and transferred into 50 µL of an LBA extraction buffer (kit ReliaPrepTM RNA Tissue Miniprep System, Promega, Madison, USA) on ice for further RNA extraction. Grape phylloxera eggs were taken and transferred onto moistened filter paper in sealed Petri dishes and incubated at 25 ± 3 °C in darkness. After three days, 800 hatched L1 larvae (N = 3 for biotype C; N = 2 for biotype A) were collected per sample in LBA buffer on ice. All insect samples were stored at −80 °C after collection.

### 2.3. RNA Extraction and RNA Sequencing Analysis

Three independent biological replicates per treatment (larval stage and biotype) were collected. An exception was grape phylloxera biotype A, stage L1, with two biological replicates. RNA extraction from whole insects was done using the ReliaPrepTM RNA Tissue Miniprep System for fibrous tissues (Promega, Madison, WI, USA), adding 40 U/µL of Ribolock (Thermo Scientific, Vienna, Austria). Extracted RNA samples were processed with the RNA 6000 Nano Kit following the manufacturer’s instructions (Agilent Technologies, Santa Clara, CA, USA). The RNA quantity and quality parameters were determined with the 2100 Bioanalyzer (Agilent Technologies Santa Clara, CA, USA). mRNA library preparation was performed with 1 μg of total RNA per sample using the TruSeq RNA Sample Prep Kit v2 according to the manufacturer’s instructions (Illumina, San Diego, CA, USA). Sequencing was performed with an Illumina HiSeq 2500 platform at the Next Generation Sequencing facility at the VetCORE-Transcriptomics unit of the VetMedUni (Vienna, Austria). An average of 47.9 M 125-bp paired-end reads was generated per sample (Table S1). Trimming for quality and length were performed with Trimmomatic, version 0.36 [50]. Reads were aligned against the reference grape phylloxera genome, using the 3.2 version, downloaded from the AphidBase platform [44] and aligned with Hisat2software, version 2.1.0 [51] with default parameters. Aligned reads were counted with HTSeq-count (version 0.9.1) in intersection-non-empty mode for overlap resolution [52] using the latest version of the gff3 file (OGS3.2_20180216). Differentially expressed genes (DEGs) analyses were performed with the R package DeSeq2 [53]. The functional gene annotation was retrieved from the BIPAA platform (v3.2), and we further performed a gene enrichment analysis using a generic Gene Ontology (GO) slim approach. Overrepresented genes categories were identified with the BINGO app 3.0.3 of Cytoscape 3.7.2 using a hypergeometric test and a significance threshold of 0.05 after Benjamini and Hochberg false discovery rate correction [54]. All raw transcriptomics reads were deposited in the NCBI Sequence Read Archive (http://www.ncbi.nlm.nih.gov/sra) with BioProject: PRJNA592030.

### 2.4. Effector Candidate Characterization and BlastP Analysis

A combined in silico secretory prediction pipeline was applied to the two subsets of upregulated genes detected for L2-3 larvae of biotype A and C [42]. The pipeline consisted of: TMHMM Server 2.0 [55], PredGPI [56], SignalP 5.0 Server [57], and SecretomeP Server 2.0 [58] applied in stepwise fashion. The subcellular localization analysis was conducted using the software tool WoLF PSORT (organism type: Animal) [59]. The functional domain analysis was conducted using Pfam (version 32) [60]. The local BlastP analysis against published aphid effectors: *A. pisum* [61,62], *M. persicae* [63], and *S. avenae* [64] was performed on the subsets of predicted grape phylloxera effectors higher and uniquely expressed in biotype A and C with NCBI Blast+ tool. Grape phylloxera BlastP matches, having an e-value < e^−50^ and a bit score > 100, were considered homologous effector candidates with those of the analyzed aphid species.

## 3. Results

### 3.1. Biotype Confirmation

Two grape phylloxera single founder lineages were tested to confirm their host performances on *V. vinifera* cv. Riesling and the rootstock Teleki 5C using the isolation chamber system. Biotype A larvae failed to form root galls on Teleki 5C, while on Riesling they successfully established 142.97 root galls per gram root dry weight (galls gDW^−1^) at 60 days after infestation (dai). Biotype C larva established root galls on both host plants. However, significantly more root galls were counted on Teleki 5C with 87.40 root galls g-1 DW than on Riesling with 9.40 root galls g-1 DW at 60 dai (Figure 1). There was no difference regarding the distribution of the root gall sizes between the treatments, except for the absence of all gall size categories in the biotype A—Teleki 5C combination.

### 3.2. Differentially Expressed Genes between Probing and Feeding Larvae

**Figure 2:**
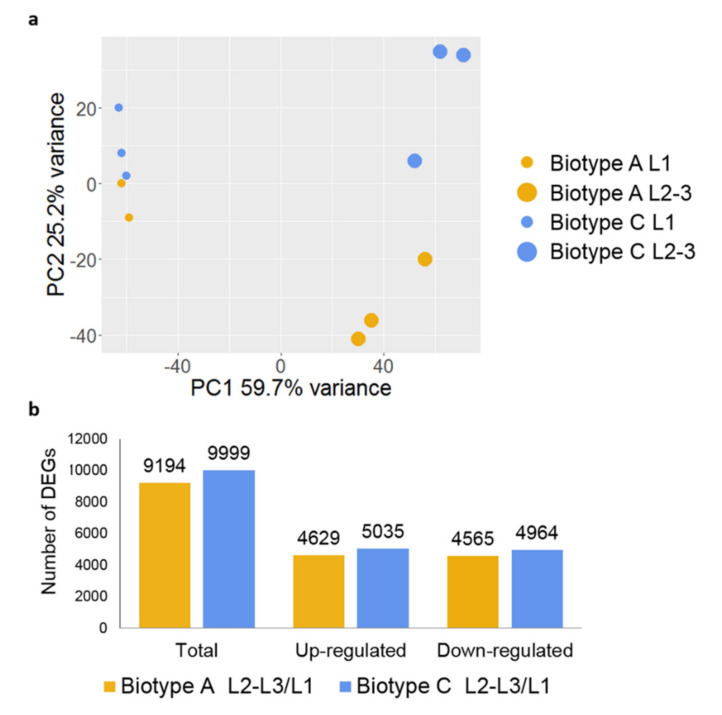
(**a**) Principal component analysis between the phylloxera transcriptome samples collected from biotype A (yellow) and C (blue) in phylloxera larval stage L1 (small circle) and L2-3 (big circle); (**b**) Differentially expressed genes in the two biotypes in the phylloxera larval stage L2-3 compared to L1. Yellow and blue colors represent biotype A and C, respectively.

Based on the transcriptional profiles, a high amount of the variance among samples could be explained by the larval stages (Figure 2a, PC1 59.7%), whereas the second component (Figure 2a, PC2 25.2%) did partially separate biotype A feeding on the susceptible host Riesling and biotype C feeding on the tolerant host Teleki 5C, especially in L2-3 stages. The comparison of larval stages within both biotypes leads to a high number of DEGs. In total, 9194 DEGs, of which 4629 were upregulated and 4565 were downregulated in L2-3 larvae in biotype A; 9999 DEGs, of which 5035 were up- and 4964 downregulated for biotype C (Figure 2b; File S1a).

**Figure 3:**
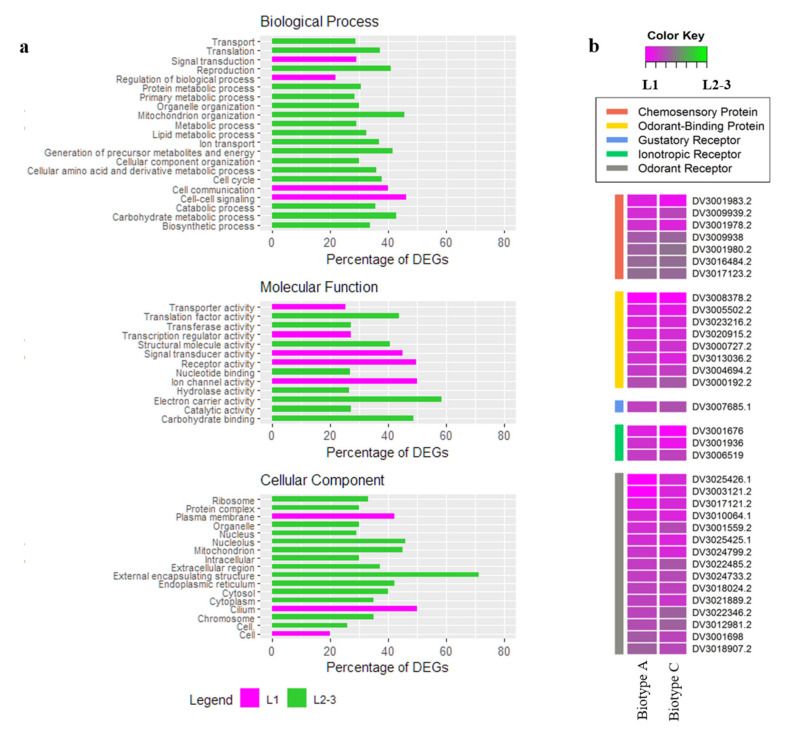
(**a**) GO-enriched categories, divided into biological process, molecular functions, and cellular component, in the DEGs between biotypes. Magenta and green bar plot colors refer to genes that are upregulated in L1 and L2-3, respectively. (**b**) Genes differentially expressed belonging to the chemosensory protein, odorant-binding protein, ionotropic receptor, odorant receptor, and gustatory receptors are represented in a heat map. Values are presented as the log_2_FC (L2-3/L1) in biotype A feeding on *V. vinifera* Riesling and C feeding on Teleki 5C.

We observed that 7501 DEGs were commonly modulated during larval development. In particular, 3576 genes were higher expressed in the L1 larvae (File S1b), whereas 3925 were expressed higher in feeding L2-3 larvae (File S1c). A GO enrichment analysis was performed by applying a general GO slim approach (Figure 3a; File S1d). The results showed that the L1 stages (of combined biotypes) expressed a higher number of genes associated with signal transduction, cell–cell signaling, cell communication, signal transducer activity, and receptor activity categories than the L2-3 stages. We identified 7 chemosensory proteins (*DV3001980.2*, *DV3017123.2*, *DV3001978.2*, *DV3016484.2*, *DV3001983.2*, *DV3009938*, *DV3009939.2*), 8 odorant-binding proteins (*DV3020915.2*, *DV3023216.2*, *DV3004694.2*, *DV3005502.2*, *DV3008378.2*, *DV3000727.2*, *DV3013036.2*), 3 ionotropic receptors (*DV3001676*, *DV3001936*, *DV3006519*), 15 odorant receptors (*DV3018907.2*, *DV3017121.2*, *DV3024799.2*, *DV3025425.1*, *DV3025426.1*, *DV3010064.1*, *DV3021889.2*, *DV3024733.2*, *DV3018024.2*, *DV3012981.2*, *DV3001698*, *DV3022346.2*, *DV3022485.2*, *DV3003121.2*, *DV3001559.2*), and 1 gustatory receptor (*DV3007685.1*) expressed higher by the L1 larvae of both biotypes (Figure 3b) following the gene annotation provided by the grape phylloxera genome [44].

For grape phylloxera L2-3 stages, the GO analysis (Figure 3a) showed enrichment of DEGs within the biological process categories: Primary metabolic processes, cellular amino acid, carbohydrate, lipid, and protein metabolic process, and generation of precursors metabolites and energy, which included several genes of the glycolysis, tricarboxylic acid cycle, electron transport chain, and pentose phosphate pathways. The cellular component categories were enriched with DEGs associated with the ribosome, mitochondrion, nucleus, and endoplasmic reticulum, altogether reflecting a high metabolic and feed digestive activity. Of interest in aphid development are some genes, mostly described with the GO annotations cellular component organization, and structural molecule activity. Among the 94 cuticular proteins identified in the grape phylloxera genome [44], we report 60 cuticular genes expressed higher during the phases L2-3 of root-feeding larvae (File S1c), indicative for an activated larval growth and development processes.

Generally, L1 larvae expressed a high number of genes related to chemoreception and host location versus L2-3 larvae expressed a higher number of genes related to insect metabolism, growth, and plant interactions.

### 3.3. DEGs in L1 Larvae Probing on Riesling vs. Teleki 5C

In the following, we aimed to identify DEGs characteristics for the grape phylloxera that might play a role for the host–parasite interaction on roots of either host. To be more conservative and to strengthen the analysis, hereafter, we considered upregulated genes with a log_2_FC > |1| and an averaged expression value > 50 after the DESeq2 normalization.

The comparative analysis revealed 169 genes significantly higher expressed in the L1 larvae feeding on *V. vinifera* Riesling, in contrast to 15 genes significantly higher expressed in L1 larvae feeding on Teleki 5C (File S2 a/b). Among them, chemosensory protein 5 (*DviCSP5*, *DV3016484.2*) and nine predicted effector candidates, according to [44], showed significantly higher expression in L1 larvae biotype A compared to C biotype: *DV3020983.1*, *DV3025302.1*, *DV3025437.1*, *DV3007994.2*, *DV3004421.1*, *DV3010742.1*, *DV3004647.2*, *DV3003294.2*, and *DV3018457.1*, and three annotated candidates: three serine-threonine kinases (*DV3010101.2*, *DV3011120*, and *DV3023215*), a serine protease inhibitor (*DV3023226*), and a carbonic anhydrase (*DV3017877*) (File S2a). In biotype C L1 larvae, we identified 15 DEGs: Among them, an induced venom protease-like protein (*DV3023993*) as a potential effector candidate of biotype C (File S2b). A high number of DEGs (61(A) and 8 (C)) have a yet unknown functional annotation.

### 3.4. DEGs in L2-3 Larvae Feeding on Riesling vs. Teleki 5C

In total, 1133 DEGs were significantly higher expressed in L2-3 feeding on *V. vinifera* Riesling if compared to L2-3 larvae feeding on Teleki 5C, which showed 789 DEGs (File S3 a/c). Among them, a relevant number of DEGs were previously predicted as grape phylloxera effectors [44]. In order to further select biotype specific effector candidates, a combined in silico secretory pipeline (Table 1) was applied to identify proteins with structural secretion signals [42]. The analysis yielded in a set of DEGs for biotype A (621) and for biotype C (380) (File S3 b/d), which were further analyzed based on general insect effector characteristics [65].

**Figure 4:**
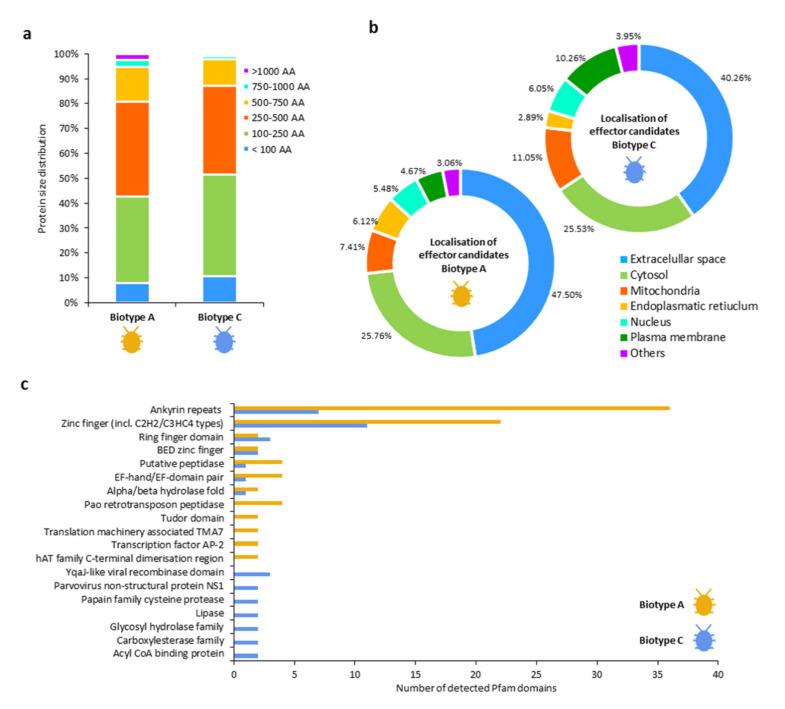
Characterization of effector candidates between feeding grape phylloxera biotype A and C, where two sets of DEGs coding for secreted proteins in the two grape phylloxera biotypes were analyzed. (**a**) Protein length distribution [AA]; (**b**) localization analysis of the grape phylloxera proteins within the insect. The results corresponded to the cellular compartment with the highest localization probability of each analyzed protein predicted by WoLF PSORT; (**c**) results of the Pfam analysis detecting functional protein domains between biotypes.

The protein length analysis showed that 94.7% of the significantly higher expressed genes of biotype A and 97.6% of biotype C coded for proteins shorter than 750 AA (Figure 4a). Using subcellular localization analysis with WoLF PSORT software, we predicted for 47.5% of DEGs in biotype A and 40.3% of biotype C that the proteins are localized in the extracellular space (Figure 4b). Seven functional protein domains commonly expressed in both grape phylloxera biotypes were detected by Pfam analysis: ankyrin repeats, zinc finger, RING finger, BED zinc finger, putative peptidase, EF-hand/EF-domain pair, and alpha/beta hydrolase fold domains (Figure 4c). Among the significantly higher expressed genes of L2-3 larvae in biotype A, we found pao retrotransposon peptidases, tudor domains, translation machinery-associated TMA7, transcription factor AP-2, and hAT family C-terminal dimerization regions. In larvae of biotype C, we identified YqaJ-like viral recombinase domains, parvovirus non-structural protein NS1, papain family cysteine proteases, lipases, glycosyl hydrolase family, carboxylesterase family, and acyl CoA binding protein domains being significantly higher expressed in L2-3 larvae as compared to the larvae of biotype A (Figure 4c).

A BlastP analysis comparing the two sets of DEGs coding for secreted proteins previously identified by the in silico pipeline (File S3 b/d) with published effector candidates of *A. pisum* [61,62], *M. persicae* [63], and *S. avenae* [64] yielded 32 additional effector candidates of biotype A and 27 of biotype C within L2-3 larvae (File S4 a/b). Within this group of effector candidates, we found additional potential effector candidates annotated as calmodulin, carbonic anhydrases, glucose dehydrogenases, serine-threonine phosphatases expressed by L2-3 larvae of biotype A and carboxylesterases, carboxypeptidases, glucose dehydrogenases, peroxidases, serine proteases expressed by L2-3 larvae of biotype C as well as a relevant number of DEGs lacking functional gene annotation (yet) (Table 2, File S3 b/d).

## 4. Discussion

### 4.1. Specific Genes Involved in Larval Chemoreception

For motile L1 larvae (as compared to sedentary feeding larvae), we report increased expression levels of chemosensory proteins (CSPs), odorant-binding proteins (OBPs), ionotropic (IRs), olfactory (ORs), and gustatory receptors (GRs) (Figure 3b). The ability to recognize, discriminate, and respond to external stimuli via their chemoreception system plays a critical role in the selection of habitats, oviposition sites, mating partners, potential predators, and host selection of insect species [66,67]. ORs are among the largest multigenic families that vary considerably between insect orders, polyphenic and developmental life stages, as well as olfactory tissues (e.g., antennae or maxillary palps) of a given insect species [66,68]. Previous analyses stated similarities in the evolutionary development between the OBPs and CSPs of grape phylloxera larvae and those of aphids involved in the perception of alarm pheromones (*DviOBP1/5/8*), host-seeking (*DviOBP2*), and the combined perception of sex pheromones and plant volatiles (*DviCSP1/2/8*) [47]. Here, we confirm the expression of 15 annotated ORs [44] that are expressed higher by probing larvae as compared to sedentary grape-feeding phylloxera larvae (Figure 3b) on both host plants. We showed differences among the biotypes in chemoreception, indicated by the significantly higher expression of *DviCSP5* (*DV3016484.2*) in biotype A L1 larvae feeding on *V. vinifera* Riesling (File S2a). *DviCSP5* was shown to be involved in host recognition processes, demonstrating its superior binding capacity to plant-released volatiles reported for the cotton bollworm *H. armigera* and the Japanese pine sawyer *M. alternatus* [69,70]. Thus *DviCSP5*—among other chemosensory proteins—may be involved in the *V. vinifera* host root selection of L1 larvae. The feeding-mode (root vs. leaf) may generally affect the number of DEGs expressed, as shown in a previous transcriptomics study [40]. Biotype A, feeding on *V. vinifera* Riesling, is a prevalent root-feeding lineage and has been reared on roots for several years in contrast to biotype C feeding both on root and leaves of Teleki 5C and being reared on both organs in the last years. The long-term feeding mode may affect the number of DEGs and even the effector profile in grape phylloxera.

### 4.2. DEGs Associated with Putative Effector Candidates Involved with Host Plant Physiology

We presented two sets of differentially expressed putative effector candidates of root-feeding grape phylloxera biotype A feeding on *V. vinifera* Riesling and C feeding on Teleki 5C (File S3 b/d), respectively. Our study confirmed 374 DEGs coding for predicted effector candidates in biotype A, and 183 DEGs in biotype C as annotated in [44]. Clearly, the host plant and feeding site, in addition to the genotype of the grape phylloxera lineages, does affect the expression of effectors or putative effector candidates (e.g., [43,71]). Several studies have been recently conducted to elucidate the grape phylloxera–host plant interaction and putative effectors involved (Table 3). The studies change in focus (e.g., leaf vs. root feeding, host plants), but they are all linked by effector function groups. The majority of grape phylloxera effector candidates were related to target host defense suppression either by the interference with early signal cascades (e.g., calreticulins, glucose dehydrogenases, heat shock proteins) or the counteracting of activated host defense mechanisms (e.g., peroxidases, peroxiredoxins) [40,41,42,43,44]. Other grape phylloxera effector candidates were thought to be functionally related to plant gall formation traits (calmodulins, calreticulins, and proteins with RING-type zinc finger, EF-hand, ankyrin repeat domains) and feed intake/digestion processes (e.g., carboxypeptidases, serine proteases, glucose dehydrogenases) [40,41,42,43,44].

However, the question rises whether effector profiles show changes responding to multiple factors, including insect genotype, life stage, host species, and host tissue type, as shown for aphids [72,73,74]. As one key finding, this study demonstrated the diversity of the effector profiles secreted by two grape phylloxera genotypes feeding on their respective *Vitis* host. A study that tests their reciprocity, like biotype A feeding larvae on Teleki 5C and biotype C feeding larvae on *V. vinifera* Riesling is underway and will allow the differentiation of effector expression linked to biotype and host plant, among other information of the host plant effect on the expression of grape phylloxera effectors. The summarized results of precious effector studies (Table 3) underline the diversity of *Vitis* hosts used for phylloxera effector studies on either leaves or root tissue and insect performance parameters chosen. Due to methodological ambiguity across grape phylloxera studies, differentiation between conservative and host/tissue type-specific effector candidates is difficult. At this stage, calreticulins, calmodulins, heat shock proteins, and proteins with RING-type zinc finger, EF-hand, and ankyrin repeat domains are consistently reported effector candidates across grape phylloxera stages feeding on leaf and root galls of different *Vitis* spp. and might therefore be considered conservative effector candidates. We found an enrichment of ankyrin repeat (43) and zinc finger (33) domains within the two sets of putative effector candidates. Both types of domains are ubiquitously found across bacterial, archaeal, and eukaryotic proteins and are reported to act as transcriptional initiators, cell cycle regulators, ion transporters, and signal transducers [75]. Expression profiles of effectors and among them an EF-hand domain effector candidate have been previously discussed as critical factors for host adaption of leaf-feeding grape phylloxera populations on different *V. riparia*, *V. arizonica*, and Frontenac host plants [43]. Here, we report the identification of two EF-hand proteins differentially expressed by the analyzed root-feeding grape phylloxera biotypes: *DV3018281.2* and *DV3021431* for biotype A; *DV3019953* for biotype C that show host-specific expression among *V. vinifera* and Teleki 5C. Biotype-specific expression was also found among effector candidates with functional RING finger domains: *DV3016877* for biotype A and *DV3001929.2*, *DV3025051.2*, and *DV3013890* for biotype C. These candidates effectively regulate microbial host–parasite interactions via the modification of ubiquitination pathways and may suppress plant defense mechanisms, such as the avoidance of programmed cell death [76,77].

**Table 3:** Current overview of the grape phylloxera–*Vitis* interactome. The table reviews previous scientific studies targeting the grape phylloxera effectors taking into account the methodological diversity of the employed insect and plant material. The first column contains the grape phylloxera effector candidates, including their putative functions, as stated in the published references. “Grape phylloxera” contains information about the genetic structure and the insect life stage of the analytical insect material used. The “Host tissue” column presents the plant material of the feeding insects. It differentiates between the *Vitis* species/cultivar and the tissue type (root versus leaf gall). Abbreviations: Pop = population; sfl = single founder lineage, T5C = rootstock Teleki 5C (*V. berlandieri* × *V. riparia*). * Effector candidates identified in feeding phylloxera larvae by the presented study.

Effector Candidate	Putative Function	Grape Phylloxera		Host Tissue		Reference
		Genetic Structure	Life Stage	Leaf Gall	Root Gall	
Calreticulins, calmodulins	Interference with Ca+ signaling, regulation of cell division/proliferation traits	Pop, sfl	L2/3/4 & A	Frontenac, Harmony, *V.arizonica*, *V.riparia*	Cab. Sauvignon, T5C	[42,43] *
Carboxypeptidases, serine proteases	Degradation of host defensive proteins, facilitation of amino acid uptake	Sfl	L2/3	Harmony	Cab. Sauvignon, T5C	[40,42] *
Esterases, mannosidases	Cell wall degradation and loosening, host defense detoxification	Sfl	L2/3	-	T5C	[42] *
Glucose dehydrogenases	Interference of host defense signaling (SA/JA/ET), detoxification, sugar intake	Sfl	L2/3	-	T5C	[42] *
Heat shock proteins	Co-regulation of abiotic stress pathways	Pop, sfl	L2/3/4 & A	*Vitis* spp.	T5C	[42,78]
Peroxidase, peroxidoredoxins	H_2_O_2_/ROS detoxification	Sfl	L2/3	-	T5C	[42] *
Protein disulfide isomerases, mucins	Stylet sheath formation	Sfl	L2/3	-	T5C	[42]
Proteins with RING-type zinc finger, EF-hand, ankyrin repeat domains	Modulation of cellular growth/development processes, ETI signaling	Pop, sfl	L2/3/4 & A	Frontenac, Harmony, *V.arizonica*, *V.riparia*	Cab. Sauvignon, T5C	[41,43,44] *
RING-containing E3 ligases	Interaction with cellulose biosynthesis (*VviCSLD5*), protein translation (*VviRPS4B*)	Pop	L2/3/4 & A	*V. riparia*	−	[41]

### 4.3. DEGs Associated with Gall Formation Traits

A significantly higher expressed gene (*DV3017805*) coding for calmodulin has been identified in larvae feeding on *V. vinifera* Riesling. Calcium-binding proteins, such as calmodulins or calreticulins, are known to affect the plant–pest interaction and were recently reported to be essential effector proteins injected by insects [79]. The capacity to encounter sieve tube occlusion via the injection of calcium-binding molecules is a controversially discussed factor allowing phloem feeding by aphid species [80,81]. Although aphids and grape phylloxera share a similar piercing-sucking feeding behavior, grape phylloxera larvae do not directly puncture phloem vessels but depend on redirecting flow from the phloem and accompanying cells towards the feeding site. Modifications of the cellular structure of phylloxerated roots have been shown to affect the expression levels of different expansins [37] and modifying the cell wall structure to enforce symplastic transfer [13]. The release of calmodulins and calreticulins by grape phylloxera, as presumed by this and other studies [42,44], may likely play an important role for the apo- and symplastic intercellular transport in order to ensure the uptake of nutrients from the surrounding root cell tissue or to maintain a certain influx pressure gradient towards the sucrose-importing phloem tissues. Alternatively, the secretion of calcium-binding molecules was associated with the suppression of early danger signaling cascades commonly employed by parasitic plant and blood-feeding insects [82]. Other studies suggest that calmodulins co-regulate cell division and proliferation events in plant tissues, thus enabling root gall formation as shown for the parasitic root nematodes *M. incognita* infecting *A. thaliana* [83,84] and may indicate a role in grape phylloxera gall induction.

### 4.4. DEGs Associated with Host Defense Response and Insect Digestive Traits

Two carboxylesterases (*DV3000733.2*, *DV3025271.1*) and one peroxidase (*DV3022019*) were shown as DEGs in biotype C. Previous studies showed that carboxylesterases are host-specifically expressed given by an increased need to detoxify and/or neutralize plant-derived toxins released from resistant host genotypes, as shown for the brown planthopper (*N. lugens*) feeding on rice cultivars (*O. sativa*) [85]. Similarly, peroxidases within the salivary secretions of grain aphids (*S. avenae*) encounter the accumulation of secondary phenolic compounds suggested to facilitate insect feeding in plant tissues [86]. Employment of neutralizing enzymes by grape phylloxera was provided [8], demonstrating the compartmentation of peroxidases, aminopeptidases, and acidic phosphatases around the stylet insertion zone. Neutralizing enzymes are thought to convert secondary plant defensive metabolites into less toxic substances [87]. Previous studies investigating the activation of the host defense pathway in grape phylloxera at different gall developmental stages point out the inductions of the phenylpropanoid, lipoxygenase, mevalonate, and isopentenyl pyrophosphate pathways, resulting in the accumulation of phenols (lignin, lignan, dihydroflavonols, anthocyanins), terpenoids (beta-caryophyllene, geraniol, beta-myrcene), and C_6_-compounds (hexanal, 2-hexenal) in gall tissues [10,15]. Gall tissues of tolerant rootstocks (preferred hosts of grape phylloxera biotype C) show the accumulation of tannins (catechin, epicatechin, and epicatechin gallate) and stilbenes (*trans*-resveratrol) increasing over time, not affecting the infestation success or fertility of grape phylloxera [16]. Based on these studies, we can conclude that grape phylloxera larvae are exposed to a wide range of plant-derived secondary metabolites involved in plant defense that vary compositionally and quantitatively depending on the host-genotype and gall developmental stage. Further studies will show if grape phylloxera biotypes and specific larval stages adapt their effector expression profiles according to the plant tissue [88] to increase levels of detoxifying and scavenging enzymes expressed by feeding larvae of biotype C on rootstocks.

Grape phylloxera feeding triggers the activation of the salicylic acid pathway [17] and the associated induction of the host defensive WRKY transcription factors in infested root gall tissue [89]. Five WRKY transcription factors (*VviWRKY72/41/33/45/46*) were shown to be higher expressed by the rootstock cultivar 140Ru as compared to *V. vinifera* cv. “Crimson Seedless” under grape phylloxera attack, implying an increased defense potential of grapevine rootstock cultivars [18]. In the presented study, we did not detect biotypic-specific grape phylloxera effector candidates with direct interaction to the *Vitis* WRKY transcription factors. More targeted research is needed to investigate the interactions between putative grape phylloxera effector candidates and the multilayered host plant defense system employing an experimental design to test reciprocal host plants.

We further report increased expression of genes encoding for carboxypeptidases (*DV3007270*) and serine proteases (*DV3020836*, *DV3020837*) by larvae feeding on Teleki 5C. Carboxypeptidases and serine proteases represent protein cleavage enzymes primarily involved in digestive processes within the insect’s gut, crucial for the nitrogen supply of developing larvae [90]. Host plants combat insect infestation by the biosynthesis of proteinase inhibitors or lectins, which, once taken up by the feeding insect, impair its digestion via the inhibition of the protein cleavage enzymes [91,92]. As a co-evolutionary response, insect genotypes developed carboxypeptidases and serine proteases insensitive to plant-derived inhibitors [93,94]. Although not yet known in grapevine, proteinase inhibitors may play a role in the defensive mechanisms against root-feeding grape phylloxera and other herbivores in rootstocks. Besides their discussed role to interact with plant defense mechanisms, the surplus of carboxypeptidases and serine proteases might increase the uptake and digestion rates of amino acids of the feed, thereby promoting larval growth and development or adult reproduction. For instance, the number of carboxypeptidase transcripts in the midgut was stimulated by the feed intake of the mosquito A*. culicifacies* [95]. Inducing a gall, as a prerequisite of the compatible grape phylloxera–*Vitis* interaction, requires the regulation of the plant nutrient allocation network to benefit the insect and to sustain the growth of the host plant. The root gall is significantly enriched in carbohydrates and amino acids [8,9] and exhibiting sink activities [13,96] to allocate carbohydrates from the plant’s sources. Sucrose import is being accelerated from phloem and subsequently transported symplastically towards the feeding site of grape phylloxera [13]. At the same time, starch granules are sequestered in the root galls. Comparing feeding with probing larvae, we found enzymes associated with intaking and digestion, such as alpha-N-acetylgalactosaminidase (*DV3006395*, *DV3010059*), glucosidases (*DV3001585*), lactase-phlorizin hydrolases (*DV3007824*), lysosomal alpha-mannosidases (*DV3009362*, *DV3016723*), maltases (*DV3005150*), and trehalases (*DV3008300*) associated with carbon metabolisms. Further, more protein cleavage enzymes (aminopeptidases (*DV3000933*), carboxypeptidases (*DV3007270*, *DV3017719*), cysteinases (*DV3000823*, *DV3015309*), amidases (*DV3001525*, *DV3017323*), and proteases (*DV3010163.2*, *DV3016734*, *DV3023272*, *DV3006797*, *DV3006796.1*) were shown to be upregulated in the feeding larvae facilitating nutrient uptake and thereby sustaining the influx gradient. We did not find biotype-specific DEG associated either with manipulation of the plant-based primary metabolisms, not directly affecting the efficacy of nutrient retrieval or digestion among the biotypes and host combinations tested in our study. Manipulation of the primary metabolism has been shown in the interaction between *M. persicae* and celery involving an aphid glutamine synthase [87,97].

At this stage, we described and discussed a selection of annotated grape phylloxera effector candidates that potentially contribute to the feeding ability on different *Vitis* spp. roots. However, other important factors play important roles for the fine-tuning of cellular processes resulting during root gall formation [98,99,100,101]. Thus, our study employing a defined bioassay exhibits part of the potential genes involved that determine host performance, in this case for biotype A and C. 

## 5. Conclusions

The presented study analyzed the transcriptional alterations comparing probing (L1) and feeding (L2-3) stages between two grape phylloxera biotypes. Our data provide the first descriptive list of key genes differentially expressed between biotypes of a root-galling insect, potentially involved in larval host plant selection between root tips of *V. vinifera* Riesling and the rootstock Teleki 5C (*V. riparia* × *V. berlandieri*) belowground. In particular, signaling genes were expressed higher in the L1 developmental stage where the mobile insect is looking for a suitable infection site, while structural developmental genes were more expressed in an advanced developmental stage (L2-3). We also observed biotypes specificity intra grape phylloxera in L2-3 larvae where, after an in silico secretory pipeline, we identified putative effectors specific for each biotype. Our presented results outline for the first time classes of common genes mutually expressed among the different developmental stages and provide a list of peculiar genes, even if not exhaustive, that may have critical roles in determining the susceptibility or partial resistance of diverse roots/rootstocks.

## Figures and Tables

**Table 1:** Combined secretory prediction pipeline. The table illustrates the steps applied to predict secreted proteins among the DEGs of root-galling grape phylloxera larvae of biotype A and biotype C. The online tools TMHMM, PredGPI, SignalP, and SecretomeP were used in a stepwise fashion to determine the likelihood of proteins being secreted [42].

**Secretory Prediction Pipeline**	**Biotype A**		**Biotype C**	
Up-regulated genes	1133	100.0%	789	100.0%
Transmembrane helices	186	16.4%	176	22.3%
GPI-anchors	8	0.7%	7	0.9%
**Transcripts without structural retaining signals**	**941**	**83.1%**	**609**	**77.2%**
Classical secretion pathway	447	39.5%	231	29.3%
Non-classical secretion pathway	407	35.9%	279	35.4%
**Secreted proteins**	**621**	**54.8%**	**380**	**48.2%**

**Table 2:** BlastP results showing effectors homologs conserved between two grape phylloxera biotypes (A, C) and predicted effector candidates of *A. pisum* [61,62], *M. persicae* [63], and *S. avenae* [64]. The functional annotations were retrieved from the grape phylloxera genome [44]. The match quality was characterized by the e-value. The table represented a selection of the effector homologues listed in File S4 a/b.

Grape Phylloxera Effector Candidate		Effector Candidate Homologs					
		*A. pisum*		*M. persicae*		*S. avenae*	
		Reference ID	*e-Value*	Reference ID	*e-Value*	Reference ID	*e-Value*
**Grape phylloxera biotype A**							
Calmodulin	DV3017805			Mp_O_17539_c0_seq4|m.16201	*5 × 10^−100^*		
Carbonic anhydrase	DV3001696	XP_003241827.1	*8 × 10^−88^*	Mp_J_18423_c0_seq1|m.16524	*10 × 10^−64^*	c14864_g1	*7 × 10^−88^*
Glucose dehydrogenase	DV3020319	XP_001946107.1	*6 × 10^−33^*	Mp_J_18221_c0_seq1|m.16042	*0 × 10^−00^*	c10172_g2	*2 × 10^−129^*
Serine-threonine phosphatase	DV3006248	XP_008186276.1	*3 × 10^−117^*				
	DV3009036	XP_008186276.1	*3 × 10^−102^*				
**Grape phylloxera biotype C**							
Carboxylesterase	DV3000733.2	XP_001947304.2	*9 × 10^−173^*	Mp_O_42087_c0_seq1|m.342653|	*6 × 10^−172^*	c15304_g1	*0 × 10^−00^*
	DV3025271.1	XP_001947304.2	*2 × 10^−157^*	Mp_O_42087_c0_seq1|m.342653|	*1 × 10^−156^*	c15304_g1	*0 × 10^−00^*
Carboxypeptidase	DV3007270			Mp_J_34059_c2_seq1|m.133176|	*0 × 10^−00^*		
Glucose dehydrogenase	DV3004487	XP_001946107.1	*0 × 10^−00^*	Mp_O_32734_c0_seq7|m.94545|	*0 × 10^−00^*	c10172_g2	*0 × 10^−00^*
Peroxidase	DV3022019	XP_008186762.1	*3 × 10^−170^*	Mp_F_35394_c1_seq17|m.129188|	*2× 10^−166^*	c14243_g1	*1 × 10^−161^*
Serine protease	DV3020836	XP_016663805.1	*6 × 10^−129^*	Mp_J_29613_c0_seq1|m.54328|	*6 × 10^−66^*	c13826_g1	*4 × 10^−109^*
	DV3020837	XP_016663805.1	*2 × 10^−110^*	Mp_J_29613_c0_seq1|m.54328|	*3 × 10^−62^*	c13826_g1	*1 × 10^−93^*

## Supplementary Materials

The following are available online at https://www.mdpi.com/2075-4450/11/10/691/s1, Figure S1: Schematic representation of the analyses described. Table S1: Transcriptome data metrics. File S1: Summary of differentially expressed genes (a) during development in the biotype A and C; commonly higher expressed genes (b) in L1 and (c) in L2-3; (d) results of the enriched GO analysis in L1 and L2-3. File S2: List of up-regulated genes of mobile grape phylloxera L1 in biotype A (a) and C (b). File S3: List of up-regulated genes of feeding grape phylloxera L2-3 from biotype A (a) and C (c) and list of candidate effectors after the in-silico pipeline run in biotype A (b) and C (d). File S4: BlastP results showing effectors homologs shared between the two grape phylloxera biotypes (A, C) and predicted effector candidates of *A. pisum*, *M. persicae*, and *S. avenae*.
