# Supplementary material for: Comparative Transcriptome Analysis of Two Root-Feeding Grape Phylloxera (D. vitifoliae) Lineages Feeding on a Rootstock and V. vinifera
Source: Insects. 2020 Oct 12;11(10):691. doi: 10.3390/insects11100691 (PMC7601026; doi:10.3390/insects11100691)
Supplement: Supplementary file 1 [file insects-11-00691-s001.zip › insects-953022- supplementary/Supplementary_Materials-Fig_S1-S2_Table_S1.docx]

**Supplementary materials**

**Figure S1**. Biotype confirmation


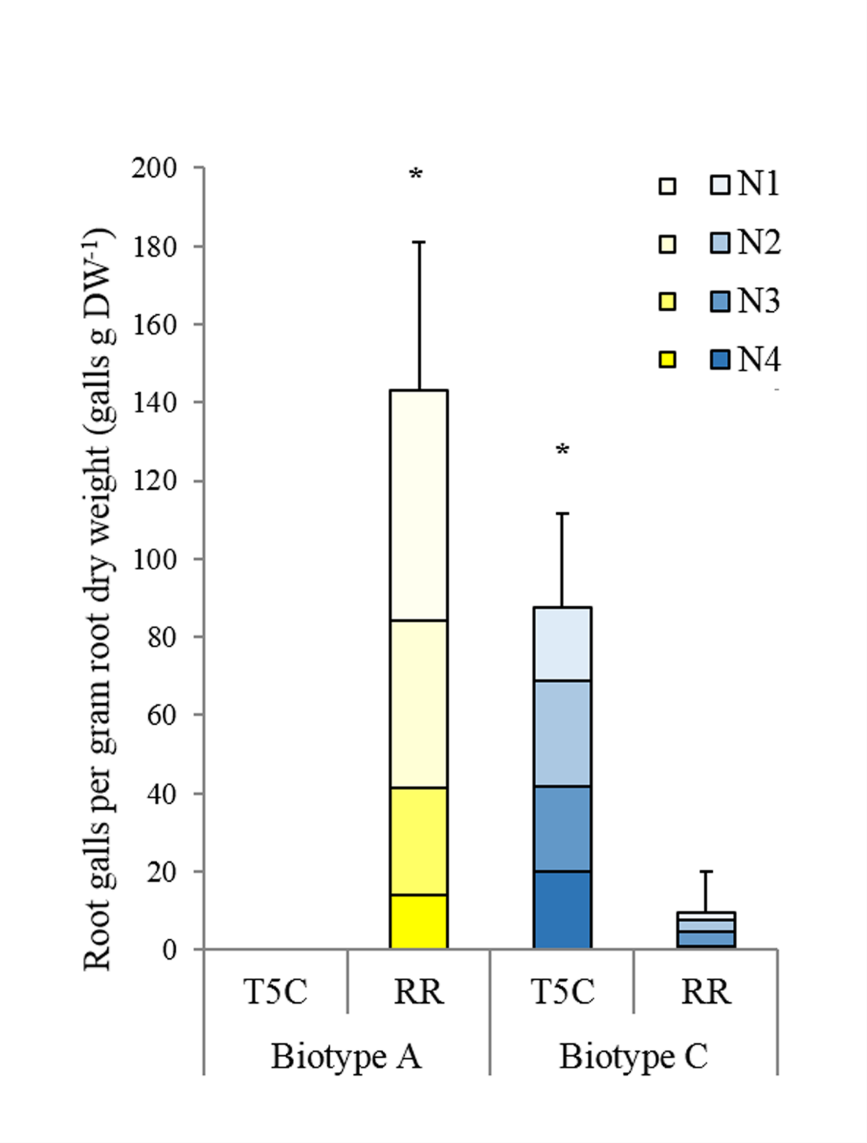


Average number of root galls (gDW-1) formed by grape phylloxera biotype A and C on roots of potted *V. vinifera* L. Riesling (RR) and the rootstock Teleki 5C (*V. berlandieri* x *V. riparia*) (T5C) 60 dai in the simple isolation chamber system [1]. Galls were categorized by size: N1 < 0.3 cm, N2 0.3 cm - 0.6 cm, N3 > 0.6 cm and N4 = inseparable galls. Error bars represented standard deviations of the sum of galls (N1-N4). Minor letters illustrated significant differences obtained by Mann-Whitney U testing with P < 0.05 comparing the sum of galls (N1-N4) produced by each biotype on the host versus the non-host.

Biotype A larva failed to form root galls on Teleki 5C, while on Riesling they successfully established 142.97 root galls per gram root dry weight (g-1 DW) at 60 days after infestation (dai). Biotype C larva established root galls on both host plants. However significantly more root galls were counted on Teleki 5C with 87.40 root galls g-1 DW than on Riesling with 9.40 root galls g-1 DW at 60 dai (Figure 2). There was no difference regarding the distribution of the root gall sizes between the treatments, except for the absence of all gall size categories in the biotype A – Teleki 5C combination.

**Figure S2** Schematic representation of the analyses described in the manuscript

**
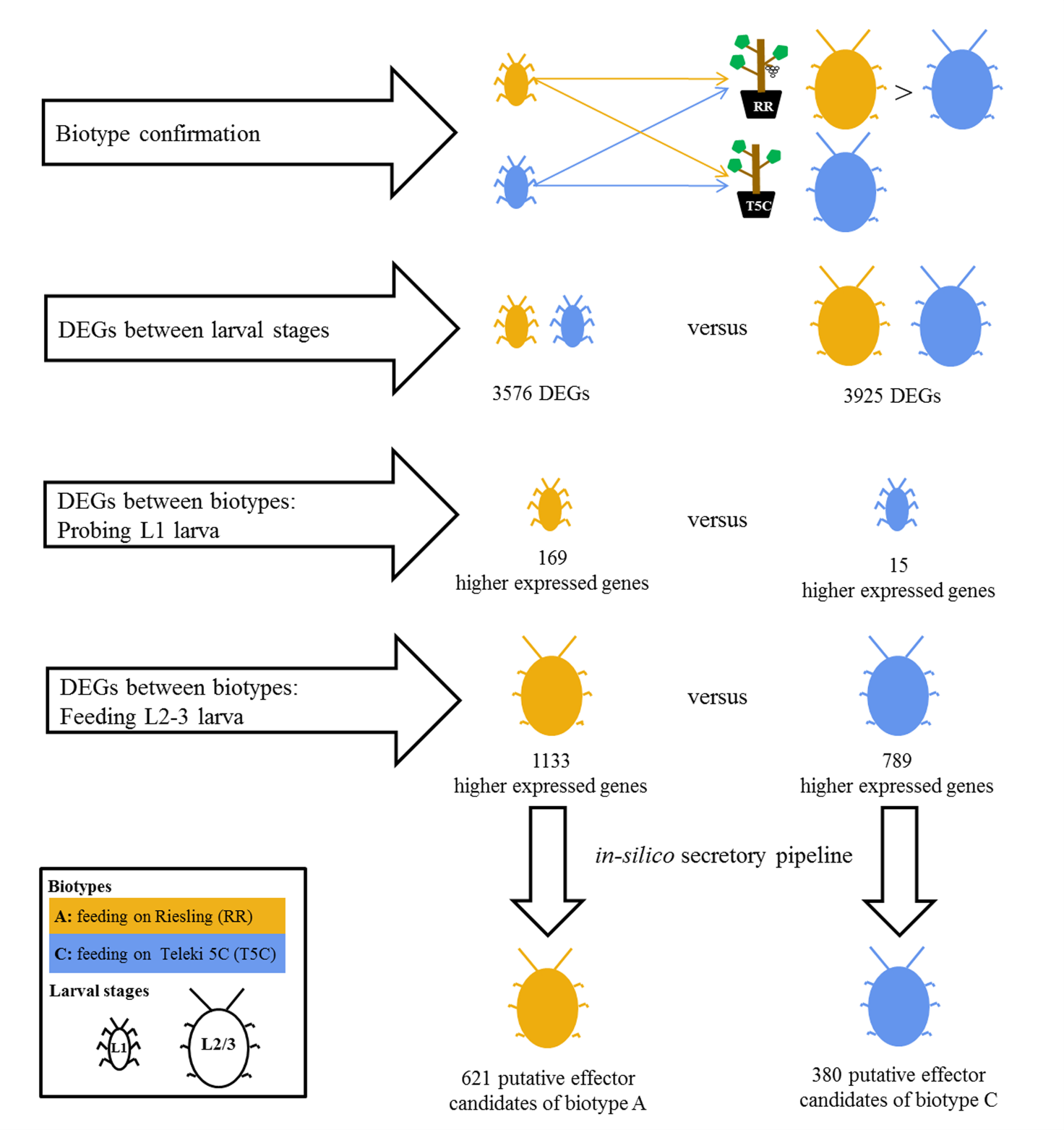
**

| **Table S1** Transcriptome data metrics. The analyses were performed in biotype A and C at phylloxera stage L1 and L2-L3 using an Illumina HiSeq platform. | | | | | | |  |
| --- | --- | --- | --- | --- | --- | --- | --- |
| **Biotype** | **Phylloxera Life Stage** | **Biol. Rep.** | **Sequenced**  **Reads** | **Trimmed**  **and Filtered Reads** | **Mapped**  **Reads** | **Unique**  **Reads** |  |
| A | L1 | 1 | 43,427,172 | 39,884,528 | 33,992,972 | 27,753,502 |  |
| A | L1 | 2 | 47,539,773 | 42,896,389 | 39,529,983 | 32,450,019 |  |
| A | L2-3 | 1 | 40,591,061 | 38,137,764 | 36,154,883 | 30,733,338 |  |
| A | L2-3 | 2 | 44,719,663 | 40,910,968 | 38,064,549 | 31,580,634 |  |
| A | L2-3 | 3 | 47,115,366 | 44,324,879 | 41,952,232 | 35,370,493 |  |
| C | L1 | 1 | 48,230,277 | 45,353,542 | 41,819,738 | 34,736,929 |  |
| C | L1 | 2 | 48,357,306 | 45,520,198 | 43,067,961 | 36,449,271 |  |
| C | L1 | 3 | 47,243,151 | 44,521,644 | 40,828,807 | 33,994,955 |  |
| C | L2-3 | 1 | 46,701,397 | 44,007,412 | 41,812,241 | 36,128,499 |  |
| C | L2-3 | 2 | 58,711,032 | 55,427,890 | 52,709,593 | 45,814,106 |  |
| C | L2-3 | 3 | 54,326,734 | 51,348,603 | 48,601,763 | 41,693,465 |  |

**Reference Supplementary Materials**

1. Forneck, A.; Walker, M.A.; Blaich, R. An in vitro assessment of phylloxera (Daktulosphaira vitifoliae Fitch) (Hom., Phylloxeridae) life cycle. *J. Appl. Entomol.* **2001**, *125*, 443–447, doi:10.1046/j.1439-0418.2001.00557.x.
