# Supplementary figures and images for: Comparative Transcriptome Analysis of Two Root-Feeding Grape Phylloxera (D. vitifoliae) Lineages Feeding on a Rootstock and V. vinifera
Source: Insects. 2020 Oct 12;11(10):691. doi: 10.3390/insects11100691 (PMC7601026; doi:10.3390/insects11100691)

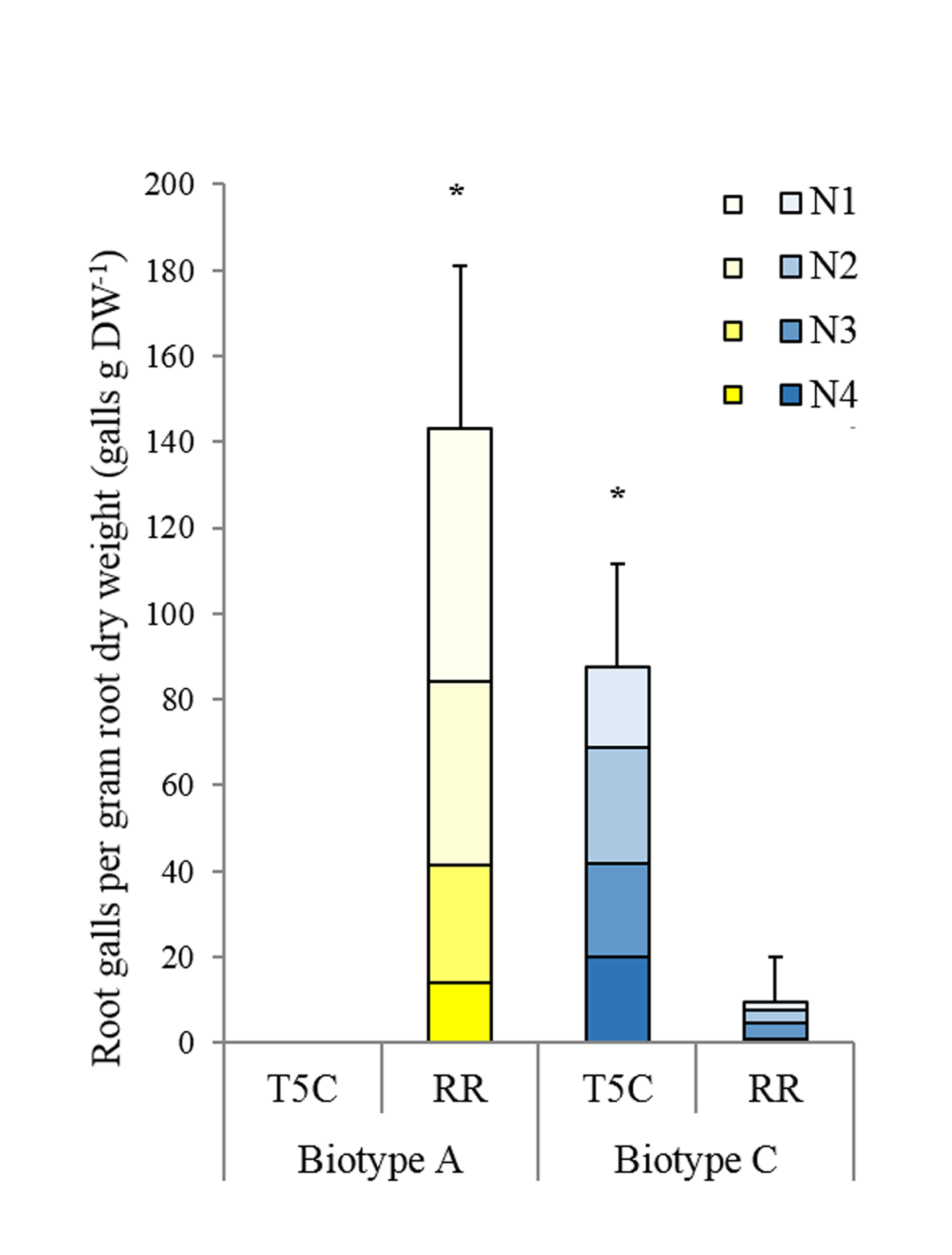

Supplement: Supplementary file 1 [file insects-11-00691-s001.zip › insects-953022- supplementary/Figure_S1.tif]

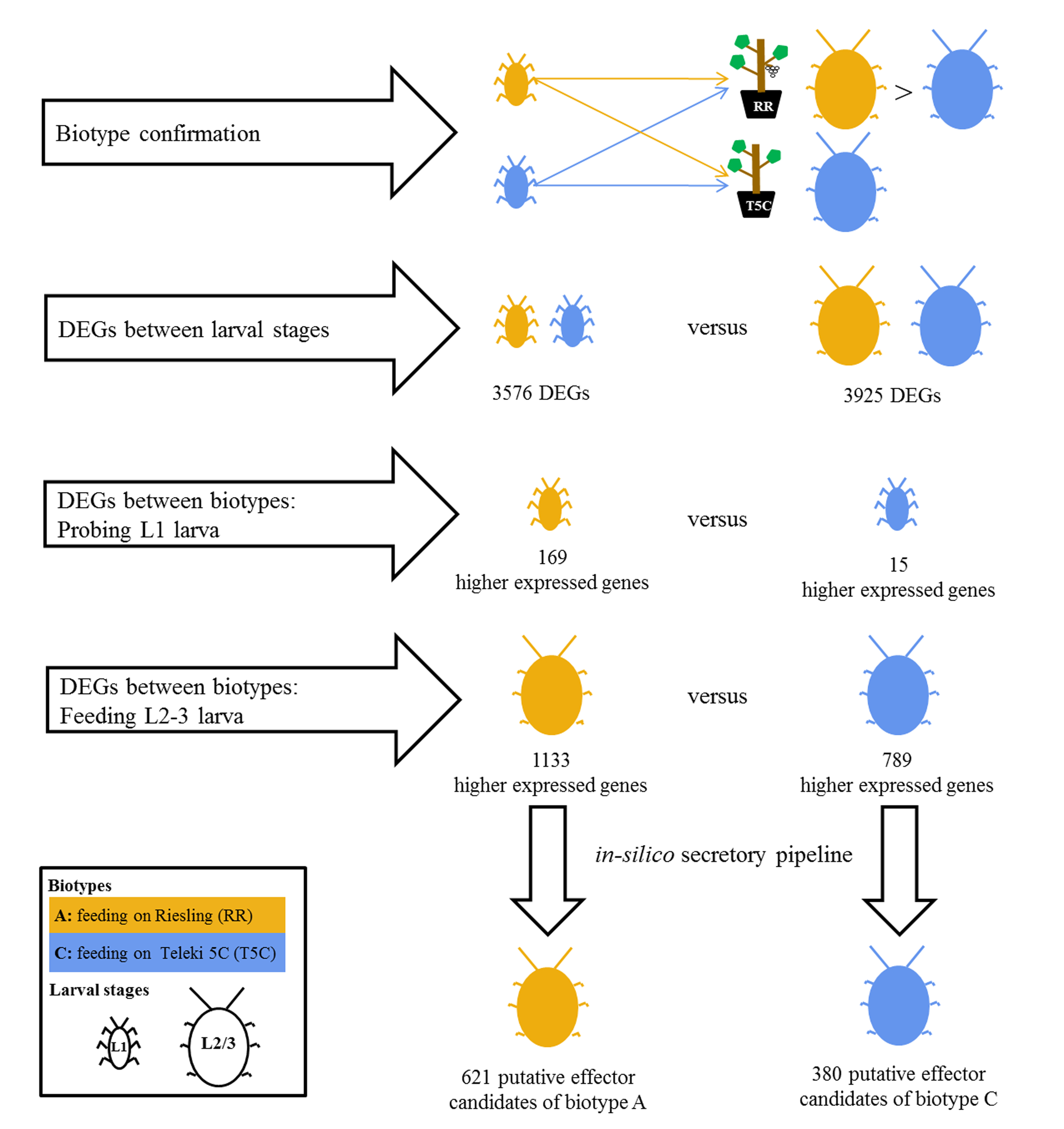

Supplement: Supplementary file 1 [file insects-11-00691-s001.zip › insects-953022- supplementary/Figure_S2.tif]
